# Improved mycelia and polysaccharide production of *Grifola frondosa* by controlling morphology with microparticle Talc

**DOI:** 10.1186/s12934-017-0850-2

**Published:** 2018-01-06

**Authors:** Ting-Lei Tao, Feng-Jie Cui, Xiao-Xiao Chen, Wen-Jing Sun, Da-Ming Huang, Jinsong Zhang, Yan Yang, Di Wu, Wei-Min Liu

**Affiliations:** 1 School of Food and Biological Engineering, Jiangsu University, Zhenjiang, 212013 People’s Republic of China; 2 National Engineering Research Center of Edible Fungi, Shanghai Academy of Agricultural Sciences, Shanghai, 201403 People’s Republic of China; 3 Jiangxi Provincial Engineering and Technology Center for Food Additives Bio-production, Dexing, 334221 People’s Republic of China

**Keywords:** *Grifola frondosa*, Microparticle Talc, Mycelial morphology, Polysaccharide biosynthesis, Enzyme activities

## Abstract

**Background:**

Mushroom showed pellet, clump and/or filamentous mycelial morphologies during submerged fermentation. Addition of microparticles including Talc (magnesium silicate), aluminum oxide and titanium oxide could control mycelial morphologies to improve mycelia growth and secondary metabolites production. Here, effect of microparticle Talc (45 μm) addition on the mycelial morphology, fermentation performance, monosaccharide compositions of polysaccharides and enzymes activities associated with polysaccharide synthesis in* G. frondosa* was well investigated to find a clue of the relationship between polysaccharide biosynthesis and morphological changes.

**Results:**

Addition of Talc decreased the diameter of the pellets and increased the percentage of S-fraction mycelia. Talc gave the maximum mycelial biomass of 19.25 g/L and exo-polysaccharide of 3.12 g/L at 6.0 g/L of Talc, and mycelial polysaccharide of 0.24 g/g at 3.0 g/L of Talc. Talc altered the monosaccharide compositions/percentages in* G. frondosa* mycelial polysaccharide with highest mannose percentage of 62.76 % and lowest glucose percentage of 15.22 % followed with the corresponding changes of polysaccharide-synthesis associated enzymes including lowest UDP-glucose pyrophosphorylase (UGP) activity of 91.18 mU/mg and highest UDP-glucose dehydrogenase (UGDG) and GDP-mannose pyrophosphorylase (GMPPB) activities of 81.45 mU/mg and 93.15 mU/mg.

**Conclusion:**

Our findings revealed that the presence of Talc significantly changed the polysaccharide production and sugar compositions/percentages in mycelial and exo-polysaccharides by affecting mycelial morphology and polysaccharide-biosynthesis related enzymes activities of* G. frondosa*.

## Background

Mushrooms provide tasty and nutritional benefits with the expanded usage to pharmaceutical and cosmeceutical areas [1, 2]. Mushroom polysaccharides are regarded as the biological macromolecules with immunomodulatory, antitumor, anti-inflammatory and hypoglycemic, and hepatoprotective activities [3]. Generally, mushroom polysaccharides are prepared from the fruiting bodies, mycelia and/or submerged fermentation broth [4–6]. In most cases, mushroom fruiting bodies grow on the solid substrates including crop by-products such as cottonseed hulls [7] and corn distiller’s waste [8, 9] for 2–4 month cultivation. Mycelia or fermentation broth is a promising alternative to meet the increasing demand on the commercial mushroom products with shorter culture period, consistent product quality and independent of seasonality [10, 11].

Similar to filamentous microorganisms, the mushroom mycelia showed pellet, clump and/or filamentous morphologies during fermentation [12, 13]. The strain physiology, culture conditions and process parameters changed morphological appearances, which further influenced the fermentation performance including nutrient consumption, oxygen uptake rate, mycelia growth and metabolites production [14]. For example, the medicinal mushroom *Ganoderma lucidum* had a twofold increase of the maximum biomass productivity with the dispersed morphology under controlled DO conditions [15]. Our previous data also revealed that high aeration (0.75–1.0 vvm) and low agitation (90 rpm) resulted in filamentous hyphae and small pellets and yielded high mycelia biomass and polysaccharide production [16].

Recently, morphology engineering strategies including targeted metabolic engineering, osmolality or operational parameter optimization have been proposed to control filamentous shape and bioprocess performance [12, 17]. Microparticle-enhanced cultivation (MPEC) such as Talc (magnesium silicate), aluminum oxide and titanium oxide was regarded as one of morphology engineering strategies to control fungal morphology development to improve productivity by preventing bulk fungal growth and providing homogenized fermentation broth [18]. For example, addition of aluminum oxide and hydrous magnesium silicate caused a dispersion of *Caldariomyces fumago* cells up to a level of single hyphae and a fivefold enhanced chloroperoxidase (CPO) production [19]. Additionally, silicate precisely adjusted the morphology of *A. niger* to a number of different distinct morphological forms [20] and Talc also led to a 50.30% increase of 3-poly-l-lysine production in *Streptomyces* sp. M-Z18 fed-batch fermentation [14].

*Grifola frondosa* is a well-known edible mushroom belonging to the order Aphyllopherales and family Polyporeceae. *G. frondosa* polysaccharides have recently attracted considerable attention for its physiological activities such as anti-tumor, hypoglycemic and immuno-stimulating properties [4, 21–23]. Our previous studies have well investigated the fermentation process optimization to maximally produce *G. frondosa* mycelia and mycelial/*exo*-polysaccharide, and elucidated the structure of mycelial polysaccharide and its antitumor mechanism [16, 21, 24]. However, relationships between fermentation conditions, mycelial growth, and polysaccharide biosynthesis need still to be further elucidated. Currently no references of microparticle-enhanced morphology engineering strategies are available to control fungal morphology and improve mycelial growth and polysaccharide biosynthesis of *G. frondosa*. Therefore, based on our previous results, the present study aimed to investigate effect of microparticle Talc addition on the mycelial growth and polysaccharide production of *G. frondosa*, and find a clue of the relationship between polysaccharide biosynthesis and morphological changes.

## Methods

### Microorganism and medium

*Grifola frondosa* GF 9801 was obtained and kept in our laboratory [24]. The strain was maintained on potato dextrose agar (PDA) after incubation at 28 °C for 7 days, stored at 4 °C and subsequently subcultured every 2 months. The seed culture was grown in a 250 mL Erlenmeyer flask containing 75 mL of the medium (glucose 20 g/L, peptone 5 g/L, KH_2_PO_4_ 1.5 g/L, and MgSO_4_·7H_2_O 0.75 g/L) at 28 °C in a shake incubator at 150 rpm for 6 days. Media for 500-mL flask fermentation with 150 mL of working volume contained glucose 60 g/L, peptone 6 g/L, KH_2_PO_4_ 3 g/L and MgSO_4_·7H_2_O 1.5 g/L.

### Inoculum preparation and shake-flask fermentation

*Grifola frondosa* was initially cultivated on PDA at 28 °C for 7 days. 10 pieces (approximately 5 × 5 mm) of the culture was transferred to the seed medium with a sterilized self-designed cutter.

To investigate the effect of Talc (3MgO·4SiO_2_·H_2_O, 45 μm) on the fermentation performance of *G. frondosa*, Talc with the concentrations of 0, 0.1, 0.5, 1.0, 3.0, 6.0, 10.0, 15.0, and 20.0 g/L was added in the 150 mL fermentation media in the 500-mL shake flasks after inoculating with 10% (v/v) of the seed. The fermentation tests were conducted at 28 °C with shaking at 150 rpm for 6 days. After removing Talc with 300 micron-size nylon mesh, the samples were collected from each flask for analyzing mycelial morphology, mycelial biomass and *exo*-polysaccharide concentrations, and polysaccharides compositions and enzymes activities related to polysaccharide-synthesis in *G. frondosa*.

### Morphological characterization

The microscopic analyses of *G. frondosa* mycelia were conducted by adding 3 mL of the culture broth into a Petri containing 10 mL of distilled water and capturing the images with a digital camera (Olympus Optical Co., Ltd., Tokyo, Japan) mounted on the stereomicroscope (Olympus Optical Co., Ltd., Tokyo, Japan). Morphological measurements were carried out using the Image-Pro Plus 6.0 software (Media Cybernetics Inc., MD, USA). The morphologies of 20 pellets in each sample were characterized by measuring the area, perimeter, equivalent diameter, circularity, and roughness [25]. Equivalent diameter is the diameter of a circle having the same area with the measured feature. It is derived from area (A) as ${\text{D}} = \sqrt {4{\text{A/}}\pi }$. According to the equivalent diameter, the mycelial pellet were divided into L-fraction (diameter ≥ 1.5 cm), M-fraction (diameter between 0.5 with 1.5 cm) and S-fraction (diameter < 0.5 cm). The circularity was estimated as the ratio of the Feret’s minimum diameter to the Feret’s maximum diameter of the pellets or aggregates. Roughness (R) was measured using the following equation: R = (pellet aggregate perimeter)^2^/(4π × pellet area).

### Fermentation performance

Fermentation performance of *G. frondosa* was evaluated based on concentrations of mycelia biomass and *exo*-polysaccharide. *Grifola frondosa* mycelia concentration was measured by centrifuging the collected broth at 10,000×*g* for 10 min and freeze-drying the precipitated mycelia after three-time distilled water wash. *Exo*-polysaccharide concentration was weighted by mixing the resulting broth vigorously with four times volume of ethanol (100%, v/v), centrifuging to obtain the precipitate at 12,000×*g* for 20 min and freeze-drying.

Mycelial polysaccharide was obtained by collecting the cultured mycelia by centrifugation at 10,000×*g* for 10 min, extracting the mycelia at 90 °C for 2 h, precipitating the extracts with four times volume of ethanol (100%, v/v) for 24 h at 4 °C, freeze-drying and weighting the precipitates. The mycelial polysaccharide yield was calculated by dividing the mycelial polysaccharide (g) by the freeze-dried mycelial biomass (g).

### Monosaccharide composition analysis of mycelial polysaccharide and *exo*-polysaccharide

Monosaccharide compositions and their ratios in mycelial polysaccharide and *exo*-polysaccharide were determined by absolute hydrolysis [26]. Briefly, Mycelial polysaccharide and *exo*-polysaccharide were hydrolyzed with 0.3 mL 72% (w/v) sulphuric acid for 30 min at 30 °C, followed with addition of 8.4 mL of distilled water and incubation at 121 °C for 1 h. The hydrolysate was neutralized with CaCO_3_, evaporated to dryness, and acetylated with Ac_2_O-Pyridine at 90 °C for 30 min. Inositol was used as an internal standard. The resulting alditol acetate was analyzed by gas chromatography using an Agilent Technologies 7890A Network gas chromatography using HP-5MS capillary column (30 m × 0.25 mm i.d.) (Hewlett-Packard, Avondale, PA, USA) with nitrogen as carrier gas. The analysis was carried out with the temperature from 130 to 240 °C at 4 °C/min and maintaining the temperature for 5 min. Samples (1 μL) were injected in the split mode at the ratio of 40:1. The products were identified by typical retention times of corresponding standards.

### Determination of PGI, UGP, UGDG, GMPPB and UGE activities associated with polysaccharide-synthesis

*Grifola frondosa* mycelia were harvested by centrifugation at 10,000×*g*, 4 °C for 10 min after 6-day fermentation, washed twice with 20 mM phosphate buffer (pH 6.5) to remove the residual media and talcum. The mycelia were ground after mixing with 20 mM phosphate buffer, and centrifuged at 10,000×*g*, 4 °C for 10 min to collect the supernatant to determine activities of the polysaccharide-synthesis related enzymes.

Polysaccharide-synthesis related enzymes including PGI (Phosphoglucose isomerase), UGP (UDP-glucose pyrophosphorylase), UGDG (UDP-glucose dehydrogenase), GMPPB (GDP-mannose pyrophosphorylase) and UGE (UDP-glucose-4-epimerase) were determined according to the previously reported method [27, 28] with slight modification. All in vitro enzyme assays were performed at 30 °C in a volume of 250 μL with 220 μL of reagents listed in Table 1, and 30 μL of cell extract. Formation or disappearance of NAD(P)H was monitored by spectrophotometrically measuring the absorbance at 340 nm (ε340 = 6.22/M/cm). The activities of the enzymes described below were expressed in nmol NAD(P)H/(mg protein)/min. The protein content of the extracts was determined using the Bradford method [29] and was compared with a bovine serum albumin standard. The blank for each enzyme was the same reaction condition with cell extract after incubation at 80 °C for 20 min.

**Table 1:** Reaction mixtures for determining the enzymes activities related to polysaccharide synthesis of *Grifola frondosa*

Enzymes	Reagents	Reagent concentration (mM)	Volume (μL)
UDP-glucose pyrophosphorylase (UGP)	Tris–HCl (pH 7.8)	50.0	200.0
	Sodium pyrophosphate	4.0	5.0
	UDP-Glc	0.4	5.0
	MgCl_2_	14.0	5.0
	NADP^+^	0.4	2.0
	PGM	2.1 U	1.1
	G-6-PD	4.0 U	1.9
UDP-glucose dehydrogenase (UGDG)	Tris–HCl (pH 7.5)	100.0	200.0
	UDP-Glc	5.0	5.0
	MgCl_2_	1.0	5.0
	NADP^+^	1.0	5.0
	DTT	1.0	5.0
GDP-mannose pyrophosphorylase (GMPPB)	MOPS (pH 7.0)	100.0	187.5
	d-Glc	4.0	3.2
	Sodium pyrophosphate	1.0	1.25
	NADP^+^	1.0	5.0
	MgCl_2_	10.0	3.6
	GDP-Man	1.0	4.1
	ADP	1.0	3.0
	NDPK	5.0 U	5.0
	HK	5.0 U	5.0
	G-6-PD	5.0 U	2.35
UDP-glucose-4-epimerase (UGE)	Tris–HCl (pH 8.5)	50.0	171.4
	UDP-Gal	0.2	2.5
	MgCl_2_	5.0	6.1
	NAD^+^	0.5	2.5
	UGDG	0.015 U	37.5

### Statistical analysis

All experiments were performed in triplicates. The data were expressed as means ± standard deviations (n = 3). Data sets were evaluated by one-way analysis of variance (ANOVA). Statistical comparisons were made on the basis of the *p* value (α = 0.05) followed by Duncan’s new multiple range test.

## Results and discussion

### Effect of Talc concentration on the morphology of *Grifola frondosa*

**Fig. 1:**
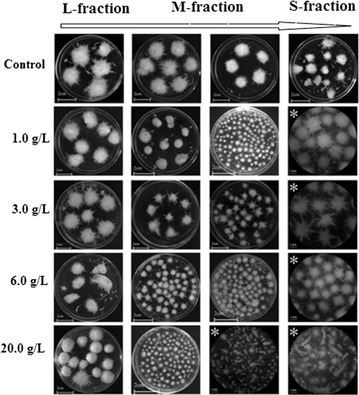
Typical morphological changes of *G. frondosa* GF9801 at different Talc concentrations ranging from 0.1 to 20.0 g/L during the 6-day fermentation period at 28 °C. (Figures having asterisk with the bar of 1 mm, and others having no asterisk with the bar of 2 cm)

Morphological forms of fungi and mushrooms vary from dispersed mycelial filaments to dense pellets which affect the amounts of secondary metabolites [30]. Our previous results also proved that *G. frondosa* mycelia had at least three typical morphological appearances including pellets with a rigid pellet core and hairy region, clumps with low roughness and filaments with uncertain shape and small size [16]. Figure 1 gave the influence of added Talc (average diameter of 45 μm) concentration on *G. frondosa* morphology during 6-day fermentation. *G. frondosa* mycelia had the bulky appearance of pellets with radial fluffs when the added Talc concentrations were 0.1–1.0 g/L. Increase of Talc concentration to 6.0–15.0 g/L decreased the rigidity and radial fluffs of mycelia and led to spherical and smooth pellets, even looser hyphal fragments. Mycelia of *G. frondosa* had the smallest size and free hypha at the Talc concentration of 20.0 g/L.

**Fig. 2:**
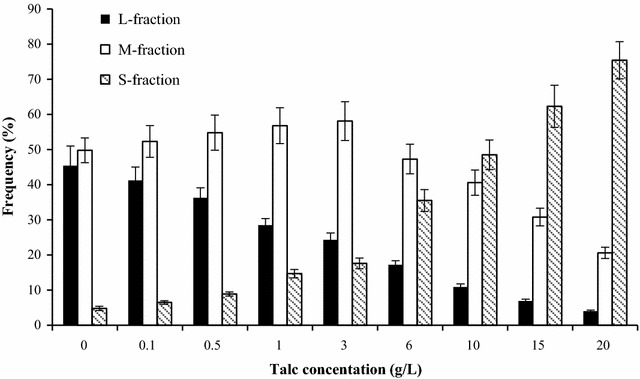
Changes of mycelial forms during submerged culture by* G. frondosa* under different Talc concentrations ranging from 0.1g/L to 20.0g/L during the 6-day fermentation period at 28 °C. (Frequency (%) = mycelia pellet No. of fraction/ total mycelia pellets× 100%; l-fraction: diameter of mycelia pellet≥1.5 cm, M-fraction: diameter of mycelia pellet ranging from 0.5 cm to 1.5 cm, S-fraction: diameter of mycelia pellet <0.5 cm)

Changes of mycelial size and its distribution during submerged culture by *G. frondosa* under different Talc concentrations were shown in Fig. 2. Majority of *G. frondosa* mycelia without Talc addition belonged to L (diameter of mycelia pellet ≥ 1.5 cm) or M-fraction (diameter of mycelia pellet ranging from 0.5 to 1.5 cm) with the ratios of 45.2 and 49.7%, respectively. The increase of Talc concentration to 20.0 g/L resulted in the significant decrease of L fraction ratio to approximately 4.12%. M-fraction ratio reached to maximum level of 58.12% when added Talc concentration of 3.0 g/L, and then gradually decreased to 20.65% with the increased Talc concentration to 20.0 g/L. Accordingly, the S-fraction (diameter of mycelia pellet < 0.5 cm) became to the majority with the maximum level of 75.41% at Talc concentration of 20.0 g/L. The present study indicated that addition of Talc also could decrease the diameters of *G. frondosa* mycelia for controlling homogeneous pellet size, which possibly due to the collision between Talc with mycelia, the shear stress exerted by the Talc and the dissociation of mycelium gathering [19]. Similar results could be found in the decrease of *Streptomyces* sp. mycelial diameter from 297.63 to 205.65 μm with supplementation of 10.0 g/L Talc [14].

Table 2 summarized the morphological parameters of *G. frondosa* S-fraction mycelia with Talc concentrations up to 20.0 g/L after 6-day fermentation. Addition of Talc with concentration of 20.0 g/L resulted in the minimum area, perimeter and equivalent diameter of 0.49 mm^2^, 4.11 and 0.79 mm, respectively, which also proved that the high Talc concentration were negative to the pellet formation. Our previous results showed that supplementation of 20.0 g/L microparticle Al_2_O_3_ decreased the area, perimeter and equivalent diameter of *G. frondosa* mycelia to 1.07 mm^2^, 6.65 and 1.17 mm [31]. Talc concentration of 3.0 g/L gave the maximum roughness of 4.86. From Table 2, Talc concentration of 0.1–15.0 g/L had a slight influence on the circularity from 0.73 to 0.81 (*p* > 0.05).

**Table 2:** Morphological parameters of pellets/mycelial aggregates during submerged culture by *G. frondosa* under different Talc concentrations

Talc concentration (g/L)	Area (mm^2^)	Perimeter (mm)	Equivalent diameter (mm)	Roughness	Circularity
0	6.75 ± 0.76a	20.25 ± 1.34a	2.93 ± 0.19a	4.83 ± 0.50a	0.73 ± 0.03a
0.1	6.23 ± 0.54ab	17.56 ± 1.43b	2.82 ± 0.24ab	3.94 ± 0.24b	0.74 ± 0.02a
0.5	5.65 ± 0.34b	14.80 ± 1.26c	2.68 ± 0.21ab	3.08 ± 0.30cd	0.81 ± 0.04a
1.0	4.75 ± 0.51c	14.65 ± 1.57c	2.46 ± 0.32b	3.60 ± 0.14bc	0.80 ± 0.07a
3.0	3.11 ± 0.23d	13.78 ± 1.24cd	1.99 ± 0.15c	4.86 ± 0.49a	0.74 ± 0.05a
6.0	3.10 ± 0.31d	11.93 ± 1.09d	1.99 ± 0.24c	3.66 ± 0.33bc	0.76 ± 0.03a
10.0	1.94 ± 0.18e	8.69 ± 0.75e	1.57 ± 0.07d	3.09 ± 0.28cd	0.79 ± 0.04a
15.0	1.62 ± 0.26e	8.33 ± 0.45e	1.44 ± 0.25d	3.40 ± 0.21bc	0.79 ± 0.08a
20.0	0.49 ± 0.05f	4.11 ± 0.38f	0.79 ± 0.03e	2.73 ± 0.18d	0.53 ± 0.01b

Each data point was the mean of three replicate samples

Roughness means the irregularity of the perimeter of pellet; Circularity represents the similarity with a circle changing from 0 to 1; a, b, c, d, e and f represent the significant difference for *p* < 0.05

### Effect of Talc concentration on fermentation performance of *Grifola frondosa*

**Fig. 3:**
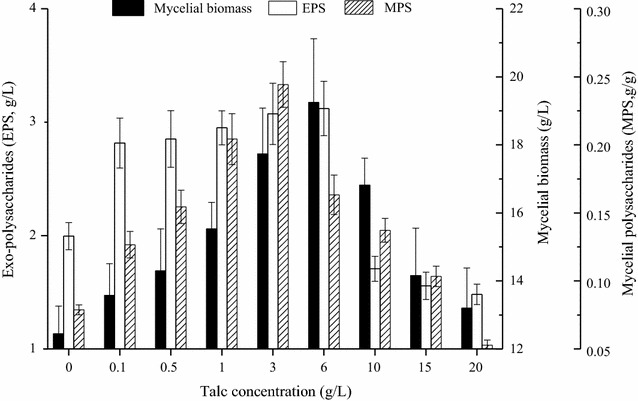
Changes of mycelial biomass, *exo*-polysaccharides (EPS) and mycelial polysaccharides (MPS) production during submerged culture by *G. frondosa* under different Talc concentrations ranging from 0.1 to 20.0 g/L during the 6-day fermentation period at 28 °C

Figure 3 showed the influence of Talc concentration on the fermentation performance of *G. frondosa*. Mycelia growth of *G. frondosa* showed an increasing trend with the increase of Talc concentrations from 0.1 to 6.0 g/L. Talc concentration of 6.0 g/L gave the maximum mycelia biomass of to 19.25 g/L with 55.0% increase to that of control. Too high concentration of Talc had the negative influence on the mycelial biomass with the minimum biomass concentration of 13.20 g/L when Talc concentration increased to 20 g/L. This finding contradicted the cultivations of *Aspergillus terreus* ATCC 20542 with highest biomass concentration at 10–15 g/L Talc [32] and *Streptomyces* sp. M-Z18 with maximum DCW of 7.05 g/L in shake-flask fermentation at 20 g L/L of Talc addition [14].

*Exo*-polysaccharide production increased to the maximum level of 3.12 g/L when the added Talc concentration increased to 6.0 g/L, and then decreased to 1.48 g/L at the Talc concentration of 20 g/L. Mycelia polysaccharide showed the highest yield of 0.24 g/g at Talc concentration of 3.0 g/L, and lowest yield of 0.05 g/g with addition of 20.0 g/L of Talc, which might contribute to the higher ratios of M- and S-mycelial fractions benefiting the heat and mass transfer. Similarly, Driouch et al. found that 10 g/L of Talc increased the 4 times of glucoamylase (GA) activity [33], and Ren et al. also found that Talc with concentration of over 10 g/L would lower the target ε-poly-l-lysine production [14]. Hence, it could be concluded that addition of 3.0–6.0 g/L of Talc would be beneficial for *exo*-polysaccharide and mycelia polysaccharide production of *G. frondosa*.

### Effect of Talc addition on monosaccharide compositions/percentages and polysaccharide synthesis related enzymes of *Grifola frondosa*

Tables 3 and 4 presented the changes of monosaccharide compositions and their ratios in the *exo*-polysaccharide and mycelial polysaccharides of *G. frondosa* without or with Talc. Similar to our previous obtained results, *exo*-polysaccharides in the control samples were mainly composed of glucose (98.25%) with minor amounts of mannose (0.89%) and arabinose (0.86%) (Table 3). Presence of Talc possibly shifted sugar compositions in the *exo*-polysaccharide of *G. frondosa*. After adding Talc microparticles with the increasing concentrations to 6.0 g/L, percentages of glucose decreased to the lowest level of 77.96% while arabinose percentage increased ranging from 2.79 to 5.43%. Interestingly, galactose appeared and reached to the highest percentage of 16.77% with adding 6.0 g/L of Talc.

**Table 3:** Effects of Talc concentration on monosaccharide composition of exo-polysaccharide isolated from *G. frondosa* GF9801

Talc concentration (g/L)	Monosaccharide compositions (%)			
	Arabinose	Mannose	Glucose	Galactose
0	0.86 ± 0.01f	0.89 ± 0.04c	98.25 ± 4.12a	–
0.1	5.43 ± 0.36a	–	94.57 ± 5.62a	–
0.5	2.83 ± 0.15de	–	93.47 ± 4.29a	3.70 ± 0.17cd
1.0	3.31 ± 0.17c	–	92.51 ± 6.13a	4.18 ± 0.32c
3.0	2.79 ± 0.11de	3.04 ± 0.20a	90.95 ± 4.81a	3.22 ± 0.16d
6.0	3.22 ± 0.34c	2.05 ± 0.21b	77.96 ± 3.54b	16.77 ± 1.32a
10.0	3.00 ± 0.22cd	0.94 ± 0.02c	90.45 ± 8.15a	5.61 ± 0.37b
15.0	3.85 ± 0.17b	0.91 ± 0.04c	90.73 ± 6.41a	4.51 ± 0.27c
20.0	2.58 ± 0.14e	0.59 ± 0.03d	96.10 ± 4.82a	0.73 ± 0.04e

Each data point was the mean of three replicate samples

a, b, c, d, e and f represent the significant difference for *p* < 0.05

**Table 4:** Effect of Talc concentrations on monosaccharide composition of mycelial polysaccharide produced by *G. frondosa* GF9801

Talc concentration (g/L)	Monosaccharide compositions (%)			
	Arabinose	Mannose	Glucose	Galactose
0	3.75 ± 0.14bc	43.03 ± 1.23bcd	50.26 ± 2.57b	2.96 ± 0.17ef
0.1	2.23 ± 0.22e	32.82 ± 1.26f	60.15 ± 4.24a	4.80 ± 0.27e
0.5	2.19 ± 0.14e	34.36 ± 1.56f	62.20 ± 4.47a	1.25 ± 0.11f
1.0	4.11 ± 0.31b	42.06 ± 2.76bcd	49.61 ± 3.25b	4.22 ± 0.23e
3.0	1.81 ± 0.09e	43.85 ± 2.54bc	43.38 ± 4.25c	10.96 ± 0.86e
6.0	6.71 ± 0.36a	62.76 ± 3.87a	15.22 ± 0.67e	15.31 ± 0.92c
10.0	3.56 ± 0.32c	40.18 ± 3.45cd	44.05 ± 2.73c	12.21 ± 0.38d
15.0	3.95 ± 0.28bc	46.40 ± 3.51b	29.57 ± 1.48d	20.08 ± 1.45b
20.0	2.76 ± 0.25d	38.10 ± 2.71de	27.95 ± 2.57d	31.19 ± 2.48a

Each data point was the mean of three replicate samples

a, b, c, d, e and f represent the significant difference for *p* < 0.05

From Table 4, Talc also significantly altered the monosaccharide compositions in mycelial polysaccharide by decreasing glucose percentage to 15.22% (6.0 g/L of Talc), and increasing galactose percentage to 31.9% (20.0 g/L of Talc) and mannose percentage to 62.76% (6.0 g/L of Talc), respectively. The previous data also proved that the aeration rate of 0.50 vvm caused the sugar metabolic shift with the lowest arabinose percentage of 2.83% and highest mannose percentage of 35.73% [16]. Similar conclusion also has been obtained that the varied dissolved oxygen concentration resulted in the metabolic shifts in *Yarrowia lipolytica* by affecting the morphological characteristics and enzymes activity such as ATP-citrate lyase in lipid biosynthesis [34].

Compared to the bacterial polysaccharides [35, 36], the knowledge about pathways and molecular mechanisms of fungal polysaccharide synthesis is still limited. Some pathways of fungal polysaccharides biosynthesis are proposed based on the monosaccharide compositions and activities polysaccharide-synthesis associated enzymes using the synthesis pathways of bacterial polysaccharide as a Ref. [27]. Based on the changes of monosaccharide compositions/percentages in *exo*-polysaccharide and mycelial polysaccharide of *G. frondosa*, the activities of five key enzymes including UGDG, PGI, UGP, GMPPB and UGE possibly involved in the polysaccharides biosynthesis were determined after 6-day fermentation and shown in Table 5. UGP and UGDG play the key roles on UDP-glucose and UDP-arabinose synthesis [37, 38]. From Table 5, UGP had the lowest activity of 91.0 mU/mg while UGDG showed the highest activity of 81.0 mU/mg with Talc concentration of 6.0 g/L, which were in accordance with the trends of glucose and arabinose percentages. GMPPB is the sole enzymes to produce GDP-mannose [39]. With the increased concentration of Talc to 6.0 g/L, GMPPB activity increased to the maximum level of 93.0 mU/mg with the corresponding mannose percentage of 62.76% in mycelial polysaccharide and 2.05% in *exo*-polysaccharide. UGE catalyzes the interconversion between UDP-glucose and UDP-galactose [40]. From Table 5, UGE activity showed the significant changes to 165 mU/mg with the increase of galactose percentage to 31.19% in mycelial polysaccharide.

**Table 5:** Effect of Talc concentrations on the enzymes activities involved in the polysaccharide biosynthesis by *G. frondosa*

Enzymatic activity (mU/mg)	Talc concentration (g/L)								
	0	0.1	0.5	1.0	3.0	6.0	10.0	15.0	20.0
PGI	632.67 ± 24.23bc	508.84 ± 20.91e	526.66 ± 31.09e	611.73 ± 15.87bcd	625.24 ± 28.74bc	782.55 ± 33.72a	588.63 ± 31.79cd	660.61 ± 34.74b	561.25 ± 43.81de
UGP	192.44 ± 10.93bc	231.53 ± 9.48a	239.67 ± 17.81a	206.78 ± 15.73b	180.67 ± 7.18c	91.18 ± 3.68e	190.24 ± 15.67bc	142.52 ± 8.28d	134.71 ± 11.32d
UGDG	64.88 ± 1.49bc	45.16 ± 2.82e	43.20 ± 1.62ef	66.26 ± 3.14b	38.91 ± 2.93f	81.45 ± 5.75a	60.28 ± 2.61c	63.71 ± 2.06bc	51.80 ± 4.19d
GMPPB	74.17 ± 2.25bc	60.54 ± 1.56d	62.37 ± 4.87d	73.76 ± 3.82bc	72.22 ± 4.58bc	93.15 ± 3.43a	70.64 ± 4.56bc	77.42 ± 6.14b	67.00 ± 3.54cd
UGE	52.72 ± 2.74f	46.66 ± 2.37f	31.54 ± 1.38g	45.81 ± 4.09f	78.45 ± 5.02e	103.27 ± 7.36c	89.32 ± 4.21d	128.26 ± 9.03b	165.11 ± 10.24a

Each data point was the mean of three replicate samples

a, b, c, d, e and f represent the significant difference for *p* < 0.05

From the above results, the possible polysaccharide synthesis pathway in *G. frondosa* could be proposed as follows. *Exo*-polysaccharide of *G. frondosa* was synthesized by converting glucose to UDP-glucose with UGP and polymerizing UDP-glucose to glucan with glucan synthases, and finally secreted to the broth. Simultaneously, glucose in *G. frondosa* cells was fermented to UDP-glucose, UDP-galactose and GDP-mannose using PGI, UGE, UGDG and GMPPB which were assembled to mycelial polysaccharides. Addition of Talc affected morphological appearance of *G. frondosa* by changing shear stress and accelerated the collision between talc with mycelia, which increased contact area with the dissolved oxygen and nutrients, altered the expression of polysaccharide synthesis related enzymes and finally changed the monosaccharide compositions in mycelial polysaccharides and *exo*-polysaccharide.

## Conclusion

In order to improve mycelial growth and polysaccharide production by *G. frondosa*, selection of microparticle Talc concentration was of highest importance. Adding Talc (45 μm) with the concentration of 3.0–6.0 g/L decreased the diameter of the pellets and increased the percentage of S-fraction mycelia of *G. frondosa*. Talc gave the maximum mycelial biomass of 19.25 g/L and *exo*-polysaccharide of 3.12 g/L at 6.0 g/L of Talc and mycelial polysaccharide of 0.24 g/g at 3.0 g/L of Talc. The presence of Talc significantly affected the monosaccharide compositions and percentages in mycelial and *exo*-polysaccharides, and enzymes activities associated with polysaccharide synthesis. The results suggested that addition of Talc would change the fermentation performance by affecting morphological appearance and polysaccharide-biosynthesis related enzymes activities of *G. frondosa*. Further investigations to construct the *G. frondosa* mutants by over-expressing and/or gene-silencing the genes involved mycelial and *exo*-polysaccharide synthesis are ongoing in our lab to enhance the production of targeted polysaccharides.
